# Effect of smoking and comorbidities on survival in idiopathic pulmonary fibrosis

**DOI:** 10.1186/s12931-017-0642-6

**Published:** 2017-08-22

**Authors:** Miia Kärkkäinen, Hannu-Pekka Kettunen, Hanna Nurmi, Tuomas Selander, Minna Purokivi, Riitta Kaarteenaho

**Affiliations:** 1 The Center of Medicine and Clinical Research, Division of Respiratory Medicine, Kuopio University Hospital, P.O. Box 100, 70029 KYS, Puijonlaaksontie 2, 70210 Kuopio, Finland; 2 Division of Respiratory Medicine, Institute of Clinical Medicine, School of Medicine, Faculty of Health Sciences, University of Eastern Finland, P.O. Box 1627, 70211 Kuopio, Finland; 3 Department of Clinical Radiology, Kuopio University Hospital, P.O. Box 100, 70029 KYS Kuopio, Finland; 4 Harjula Hospital, the Municipal Hospital of Kuopio, Niuvantie 4, 70101 Kuopio, Finland; 5 The Science Services Center, Kuopio University Hospital, P.O. Box 100, 70029 KYS Kuopio, Finland; 6 Respiratory Medicine, Internal Medicine Research Unit, Medical Research Center Oulu, Oulu University Hospital and University of Oulu, P.O. Box 20, 90029 Oulu, Finland

**Keywords:** Idiopathic pulmonary fibrosis, smoking, gender, comorbidity

## Abstract

**Background:**

Cigarette smoking has been associated with the risk of idiopathic pulmonary fibrosis (IPF). Certain comorbidities have been associated with reduced survival although some studies have indicated that current smokers have a longer survival than ex-smokers. Comorbidities in relation to smoking history have not been previously analyzed.

**Methods:**

Retrospective data was collected and patients were categorized according to gender and smoking habits. Comorbidities and medications were collected. Predictive values for mortality were identified by COX proportional hazard analyses.

**Results:**

We examined 45 non-smokers (53.3% female), 66 ex-smokers (9.1% female) and 17 current smokers (17.6% female) with IPF. Current smokers were younger at baseline (58.1 ± 8.74 years) compared to non-smokers (71.4 ± 8.74, p < 0.001) and ex-smokers (72.5 ±7.95, p <0.001). Median survival of non-smokers and current smokers was longer (55.0 and 52.0 months, respectively) than that of ex-smokers (36.0 months) (p=0.028 and 0.034, respectively). In age and severity adjusted analyses, smoking was not related to survival. Cardiovascular diseases (CVD) (72.7 %) were the most common comorbidities, current smokers had more chronic obstructive pulmonary disease (COPD) and lung cancer compared to ex-smokers (p<0.001). CVD, COPD and use of insulin were related to poorer survival in adjusted analyses.

**Conclusions:**

Smoking seems to influence the course of disease in IPF since current smokers developed the disease at a younger age in comparison to non-smokers and ex-smokers. No significant differences in the major comorbidities were detected between IPF patients with different smoking histories. The mechanism through which smoking influences IPF progression requires further investigation.

## Background

Cigarette smoking has been shown to associate with the risk of developing idiopathic pulmonary fibrosis (IPF) with ever-smokers having a 60 % higher risk [1]. There are, however, controversial reports on how smoking affects survival in IPF. Current smokers at the time of IPF diagnosis have been found to have longer survival times than ex-smokers as well as non-smokers and ex-smokers; however, if one applies the composite physiologic index (CPI) for severity adjustment, then never-smokers had longer survival times than ever-smokers (i.e. current and ex-smokers) and furthermore the survival difference between current and ex-smokers disappeared [2, 3]. A recent study found that ever-smokers with IPF lived longer than never-smokers who, however, revealed significantly higher CPI and thus severity adjusted survival remained significantly different between the two groups [4]. Males with current or past smoking histories and occupational exposure have been reported to carry an increased risk of IPF compared to non-exposed females [5].

In addition to smoking, comorbidities might influence the progression of IPF i.e. patients with several comorbidities have been shown to exhibit worse survival than those not burdened by comorbidities [6]. The most prevalent comorbidities in IPF have been shown to be pulmonary hypertension, obstructive sleep apnea (OSA), lung cancer, chronic obstructive pulmonary disease (COPD), coronary artery disease (CAD) and gastro-esophageal reflux (GER) [7]. Furthermore, IPF-patients have been reported to have an increased risk of vascular disease, diabetes and hypertension [6, 8–10]. Thyroid disease, diabetes, CAD and lung cancer are all illnesses that have been shown to associate with shortened survival in IPF, whereas the use of GER medication has been claimed to prolong patient lifespans [10–15]. The results on the use of statin and anticoagulant medications on the survival of IPF patients have been controversial [10, 16–19].

The aims of this study were to evaluate the clinical and radiological characteristics of patients with IPF in a retrospective cohort from an eastern Finnish Hospital and to subdivide them according to smoking history i.e. non-smokers, ex-smokers and current smokers in both the whole study group, and also separately for female and male patients. Furthermore, we wanted to examine the numbers and types of comorbidities and medications, and evaluate their effect on survival of the patients.

## Materials and Methods

### Patients and data collection

A total of 223 patients (91 female, 132 male) with pulmonary fibrosis treated in Kuopio University Hospital between 1^st^ January 2002 and 31^st^ December 2012 were collected from medical records of the hospital by using International Classification of Diseases version 10 (ICD-10) codes J84.1, J84.8 and J84.9. Clinical, radiological and histological information of each patient was transferred from medical records to special data collection forms designed for the present study. Pulmonary fibroses with a known etiology, i.e. connective-tissue disease, occupational exposure etc., were excluded. The data concerning comorbidities and survival was updated on 16^th^ October 2016.

Smoking status was categorized as non-smoker, ex-smoker and current smoker and collected as years, pack-years or both if available. Ex-smokers were defined as having smoked regularly for more than one year, and longitudinal changes in smoking habits after the diagnosis of IPF were not considered. The presence of comorbid diseases i.e. COPD, asthma, hypertension, heart failure for any reason, CAD, cerebral infarction, transient ischemic attacks, OSA, lung cancer, GER, depression, diabetes and other cancers were gathered from the medical records; in this case the time of diagnosis was the cut-off for subdivision into either before or after the diagnosis of IPF. Pulmonary function tests (PFT) included results of spirometry i.e. forced vital capacity (FVC), forced expiratory volume in one second (FEV1) and FEV1/FVC ratio, diffusion capacity i.e. diffusion capacity of carbon monoxide (DLco) and potential value of diffusion capacity per liter of lung volume (DLco/VA). PFT results were expressed as absolute numerical values and percentages of predicted values. Information from histological samples from surgical lung biopsy or autopsy was gathered.

No consents for inclusion into this study were gathered since this was a retrospective study and the majority of the patients are deceased. The study protocol was approved by the Ethical Committee of Kuopio University Hospital (statement 17/2013) and from the National Institute for Health and Welfare (Dnro THL/1052/5.05.01/2013). Permission to use data from death certificates was given by Statistics Finland (Dnro: TK-53-911-13). This study was conducted in compliance with the Declaration of Helsinki.

### Assessment of clinical, radiological and histological data

The PFT were evaluated using the Finnish reference values [20]. CPI was calculated from the PFT results as follows: 91.0 – (0.65 x % predicted DLco) – (0.53 x % predicted FVC) + (0.34 x % predicted FEV1) [21]. The findings of the first and last high resolution computed tomography (HRCT) of each patient were re-evaluated by a radiologist according to the recent statement of the American Thoracic Society (ATS) and European Respiratory Society (ERS) and re-classified as usual interstitial pneumonia (UIP), possible UIP and not UIP [22]. All the patients whose re-analyses were categorized as not definite UIP in HRCT were evaluated by a multidisciplinary discussion with a radiologist, a pathologist and a pulmonologist before being included into the present study. Either death or lung transplantation were considered as end-points in the survival analyses.

### Statistical analysis

Statistical analysis was performed with IBM SPSS Statistics version 21. Group differences were tested by Kruskall-Wallis or Mann-Whitney U-test for continuous variables and by Chi-square testing or Fisher exact test for categorical variables. Data is presented as mean with standard deviation for continuous variables or frequencies with percentages when variables are categorical. Survival curves were estimated by the Kaplan-Meier method and differences in survival were compared using log-rank test, in addition median survival is expressed. Univariate and multivariate survival analysis were computed using Cox regression models. Results from Cox survival analyses are shown in the form of a hazard ratio with 95 % confidence intervals. An ordered logistic regression was performed in order to evaluate the impact of age and smoking status on the number of comorbidities. P-value < 0.05 was considered as statistically significant.

## Results

### Demographics and unadjusted survival

**Fig. 1:**
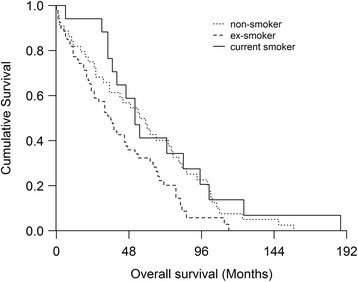
Analyses of survival indicates that ex-smokers revealed shorter survival time (36 months) than current smokers (52 months (0.029)) or non-smokers (55 months (p=0.034))

A total of 132 patients with IPF were evaluated in this study; of these, 97 (73.5%) were male and 35 (26.5%) female. The mean age of the patients at the time of diagnosis was 70.5 years and the median survival 45.0 months. When we updated the data (16^th^ October 2016), a total of 115 patients had deceased and 15 patients were still alive. Three patients (one of whom had died) had undergone lung transplantation. Two males and 2 females had an unknown smoking history. From the 128 (96.7%) patients with a known smoking history, 45 (35.2%) were non-smokers, 66 (51.2%) ex-smokers and 17 (13.4%) were current smokers. There were more female non-smokers (p <0.001) and fewer ex-smokers than their male counterparts (p <0.001) but no gender difference was seen in the proportion of current smokers (p=0.410). Current smokers had smoked for significantly more years and more pack-years than ex-smokers and they were also significantly younger at the time of diagnosis and at death compared to non-smokers and ex-smokers; this remained the case when male patients were analyzed separately. Current smoker females were younger at the time of diagnosis compared to ex-smoking females (0.036) and they died at a younger age than their non-smoking counterparts (p=0.038). Female patients had significantly longer survival time compared to males, 42.2 months and 29.1 months, respectively (p=0.039) and ex-smokers had a shorter survival time compared to current smokers and non-smokers (Table 1, Fig. 1). No significant differences in PFT results, CPI values or HRCT findings were detected between the groups of different smoking histories (Table 1).

**Table 1:** Demographics of the patients with IPF according to their smoking status

	Whole cohort	Ex-smoker (ES)	Current smoker (CS)	Non-smoker (NS)	p-value (between ES and CS)	p-value (between ES and NS)
N (%)^a^	132 (100%)	66 (50.0)	17 (12.8)	45 (34.1)		
Pack years (SD)	25.9 (17.66)	23.1 (18.43)	32.9 (13.65)		0.006	
Smoking years (SD)	25.5 (13.54)	21.8 (12.48)	38.1 (8.48)		<0.001	
Male N (%)	97 (73.5)	59 (90.8)	14 (82.4)	20 (44.4)	0.312	<0.001
Age at diagnosis (y)(SD)	70.5 (9.80)	72.4 (7.95)	58.1 (9.93)	71.7 (8.74)	<0.001	0.411
Age at death (y) (SD)	74.4 (9.35)	75.8 (8.06)	63.1 (8.13)	76.2 (8.47)	<0.001	0.877
Median survival (mo)	45.0	36.0	52.0	55.0	0.029	0.034
Pulmonary function tests (mean (SD))						
FVC% (SD)^b^	76.7 (18.51)	75.6 (19.51)	76.1 (17.48)	78.3 (17.26)	0.926	0.341
FEV1% (SD)	77.0 (16.99)	74.9 (17.64)	75.1 (15.77)	80.7 (16.26)	0.866	0.050
FEV1/FVC% (SD)	101.4 (9.68)	100.0 (9.70)	99.8 (11.84)	103.7 (7.72)	0.995	0.093
DLco% (SD)^c^	56.1 (17.51)	53.8 (16.13)	58.9 (19.49)	58.2 (18.98)	0.272	0.139
CPI (SD)	39.9 (19.96)	41.1 (13.63)	37.9 (14.25)	39.1 (14.67)	0.312	0.341
Radiological diagnoses N (%)						
Definite UIP	79 (61.7)	37 (46.8)	14 (61.7)	28 (35.4)	0.055	0.553
Possible UIP	28 (21.9)	19 (67.9)	1 (3.6)	8 (28.6)	0.049	0.261
Not UIP	21 (16.4)	10 (47.6)	2 (11.8)	9 (42.9)	0.723	0.794

Data expressed mean (SD) or N (%) of patients

*N* number, *y* years, *mo* months, *FVC* forced vital capacity, *% pred* percent predicted, *FEV1* forced expiratory volume in one second, *DLco* diffusion capacity of carbon monoxide, *CPI* composite physiologic index, *UIP* usual interstitial pneumonia, *SD* standard deviation

^a^smoking status of 4 patients (2 male and 2 female) was unknown

^b^Spirometry results from 126 patients

^c^Diffusion capacity from 124 patients

In the univariate analyses, DLco% and CPI were significantly related to survival: for DLco% hazard ratio (HR) was 0.97 with 95% confidence interval (95% CI) 0.96 – 0.98 and with p-value <0.001 and for CPI HR was 1.04, 95% CI 1.02 – 1.06 and p-value < 0.001. For that reason DLco% and CPI were used in the severity adjustment in the multivariate analyses.

### Step-by-step multivariate analyses

When survival differences were compared between ex-smokers and current smokers in step-by-step multivariate analyses i.e. adding one factor at a time to the model using DLco % and CPI in severity adjustment, the survival difference in favor of current smokers was reduced to a marginally non-significant level (p=0.098 and p=0.128, respectively). When age at the time of diagnosis was added into the multivariate analyses, smoking history no longer exerted any statistically significant effect on survival (Table 2). When survival differences were compared between ex-smokers and non-smokers, the better survival of non-smokers disappeared after severity adjustment with DLco% and CPI while age remained as a significant predictor of survival (Table 3). Male gender was found to be a significant risk factor for shorter survival when comparing ex-smokers and non-smokers, but not in the comparison between ex-smokers and current smokers (Tables 2 and 3).

**Table 2:** A comparison of survival between ex-smokers and current smokers in the step-by-step multivariate models

	HR	95% CI	*p*-value
Univariate			
Ex-smoker	Reference		
Current smoker	0.52	0.29 – 0.95	0.033
Model containing DLco%			
Ex-smoker	Reference		
Current smoker	0.60	0.33 – 1.10	0.098
Dlco%	0.97	0.96 – 0.99	0.001
Model containing CPI			
Ex-smoker	Reference		
Current smoker	0.62	0.34 – 1.14	0.126
CPI	1.03	1.01 – 1.05	0.001
Model containing DLco % and age			
Ex-smoker	Reference		
Current smoker	1.32	062 – 2.72	0.472
Dlco%	0.97	0.95 – 0.98	<0.001
Age at diagnosis	1.06	1.02 – 1.10	0.001
Model containing CPI and age			
Ex-smoker	Reference		
Current smoker	1.63	0.75 – 3.54	0.217
CPI	1.05	1.02 – 1.07	< 0.001
Age at diagnosis	1.07	1.03 – 1.12	<0.001
Model containing DLco %, age and gender			
Ex-smoker	Reference		
Current smoker	1.32	063 – 2.80	0.465
Dlco%	0.97	0.95 – 0.99	<0.001
Age at diagnosis	1.06	1.03 – 1.10	0.001
Gender - female	Reference		
male	1.10	0.45 – 2.69	0.831
Model containing CPI, age and gender			
Ex-smoker	Reference		
Current smoker	1.66	0.76 – 3.64	0.203
CPI	1.05	1.02 – 1.07	<0.001
Age at diagnosis	1.07	1.03 – 1.12	<0.001
Gender - female	Reference		
male	1.22	0.51 – 2.94	0.657

*HR* hazard ratio, *CI* confidence interval, *DLco* diffusion capacity of carbon monoxide, *CPI* composite physiologic index

**Table 3:** A comparison of survival between ex-smokers and non-smokers in the step-by-step multivariate models

	HR	95% CI	p-value
Univariate			
Ex-smoker	Reference		
Non-smoker	0.64	0.42 – 0.97	0.037
Model containing DLco%			
Ex-smoker	Reference		
Non-smoker	0.79	0.50 – 1.24	0.306
Dlco%	0.97	0.95 – 0.98	<0.001
Model containing CPI			
Ex-smoker	Reference		
Non-smoker	0.75	0.48 – 1.17	0.197
CPI	1.04	1.02 – 1.06	<0.001
Model containing DLco % and age			
Ex-smoker	Reference		
Non-smoker	0.82	0.52 – 1.27	0.371
Dlco%	0.96	0.95 – 0.98	<0.001
Age at diagnosis	1.05	1.02 – 1.08	0.001
Model containing CPI and age			
Ex-smoker	Reference		
Non-smoker	0.79	0.51 – 1.23	0.292
CPI	1.05	1.03 – 1.07	<0.001
Age at diagnosis	1.06	1.03 – 1.09	<0.001
Model containing DLco %, age and gender			
Ex-smoker	Reference		
Non-smoker	1.32	0.76 – 2.30	0.324
Dlco%	0.96	0.95 – 0.98	<0.001
Age at diagnosis	1.06	1.03 – 1.09	<0.001
Gender - female	Reference		
male	2.35	1.24 – 4.45	0.009
Model containing CPI, age and gender			
Ex-smoker	Reference		
Non-smoker	1.22	0.71 – 2.10	0.472
CPI	1.05	1.03 – 1.07	<0.001
Age at diagnosis	1.07	1.04 – 1.10	<0.001
Gender - female	Reference		
male	2.22	1.18 – 4.18	0.013

*HR* hazard ratio, *CI* confidence interval, *DLco* diffusion capacity of carbon monoxide, *CPI* composite physiologic index

### Comorbidities and medications

**Fig. 2:**
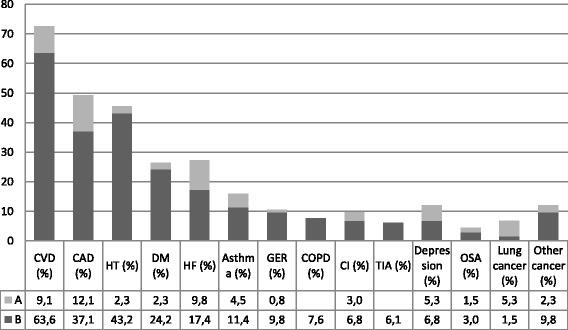
Prevalence of comorbidities in the IPF patients are presented as percent of total (N=132). The time of comorbidity diagnoses are presented before and after the diagnosis of IPF. CVD includes CAD, HT and CI. B, diagnosed before the diagnosis of IPF; A, diagnosed after the diagnosis of IPF; CVD, cardiovascular disease; CAD, coronary artery disease; HT, hypertension; DM, diabetes; HF, heart failure for any reason; GER, gastro-esophageal reflux; COPD, chronic obstructive pulmonary disease; CI, cerebral infarction; TIA, transient ischemic attack; OSA, obstructive sleep apnea. Other cancers treated before the diagnosis of IPF included seminoma, melanoma, basal cell carcinoma, renal, ventricular, prostate, bone, colorectal, breast and thyroid cancer. One patient had three different cancers. Two colorectal cancers were detected after the diagnosis of IPF

Twenty-one (15.9%) of the patients did not have any comorbidities while 36 (27.3%) had one, 30 (22.7%) had two, 20 (15.2%) had three, 21 (15.9) had four and 4 (3.0%) had five comorbidities. The most common comorbidities were cardiovascular diseases (CVDs) (72.7 %) (Fig. 2). Females were more likely than males to suffer from asthma, hypertension or diabetes. Current smokers had significantly more COPD (p=0.000) and lung cancer (p=0.006) compared to ex-smokers, this difference was observed in males, but not in females when the data was subdivided according to genders (Table 4). The multivariate analyses were adjusted for age, gender and smoking status and in addition, DLco % or CPI in two different models (Table 5). In multivariate analysis with DLco %, CVD and COPD were related to poorer survival and in the multivariate analysis with CPI, it was noted that the use of insulin was related to poorer survival (Table 5). In the ordered logistic regression, age, but not smoking history, exerted a significant impact on the number of comorbidities (standardized beta coefficient 1.044, p= 0.003), but the number of comorbidities did not have any statistically significant effect on survival even in multivariate analyses.

**Table 4:** Comorbidities of patients with IPF according to their smoking habits

	Non-smokers	Ex-smokers	Current smokers	*p*-value
Cardiovascular diseases ^a^	34 (75.6)	46 (69.7)	12 (70.6)	0.790
Coronary artery disease	22 (48.9)	29 (43.9)	10 (58.8)	0.537
Hypertension	23 (51.1)	28 (42.4)	6 (35.3)	0.474
Cerebral infarction	3 (6.7)	8 (12.1)	2 (11.8)	0.629
TIA	3 (6.7)	5 (7.6)		0.511
Diabetes	13 (28.9)	18 (27.3)	1 (6.3)	0.145
Heart failure ^b^	16 (35.6)	14 (21.2)	3 (17.6)	0.169
Asthma	7 (15.6)	9 (13.6)	4 (23.5)	0.605
Depression	4 (8.9)	4 (6.1)	1 (6.3)	0.832
GER	3 (6.7)	10 (15.2)	1 (6.3)	0.288
Lung cancer		4 (6.1)	5 (29.4)	<0.001
COPD		3 (4.5)	7 (41.2)	<0.001
OSA	2 (4.4)	3 (4.5)	1 (6.3)	0.969

*N* number, *NS* p-value > 0.05, *TIA* transient ischemic attack, *GER* gastro-esophageal reflux, *COPD* chronic obstructive pulmonary disease, *OSA* obstructive sleep apnea

^a^Coronary artery disease, cerebral infarction and hypertension

^b^Heart failure for any reason

**Table 5:** Comorbidities and their association with survival after adjustment for age, smoking status, gender and DLco% or CPI

		Multivariate with DLco%^a^		Multivariate with CPI ^a^	
	N(%)	HR (95 % CI)	p-value	HR (95 % C I)	p-value
Cardiovascular diseases ^b^	96 (72.7)	1.6 (1.01 – 2.52)	0.047	1.5 (0.94 – 2.38)	0.086
Coronary artery disease	65 (49.2)	1.4 (0.92 – 2.05)	0.124	1.4 (0.93 – 2.10)	0.109
Hypertension	60 (45.5)	1.0 (0.69 – 1.52)	0.900	1.0 (0.67 – 1.47)	0.955
Heart failure ^c^	36 (27.2)	0.8 (053 – 1.25)	0.339	0.8 (0.53 – 1.27)	0.378
Cerebral infarction	13 (9.8)	0.8 (0.40 – 1.44)	0.395	0.9 (0.45 – 1.63)	0.638
TIA	8 (6.1)	1.1 (0.48 – 2.66)	0.778	1.2 (0.52 – 2.89)	0.648
Diabetes	35 (26.5)	1.3 (0.82 – 2.11)	0.256	1.4 (1.03 – 1.07)	0.198
OSA	6 (4.5)	0.5 (0.22 – 1.20)	0.121	0.5 (0.21 – 1.59)	0.105
GER	14 (10.6)	0.9 (0.49 – 1.74)	0.799	0.9 (0.46 – 1.64)	0.661
Asthma	21 (15.9)	1.6 (0.92 – 2.78)	0.098	1.5 (0.89 – 2.66)	0.126
COPD	10 (7.6)	2.5 (1.02 – 6.01)	0.045	1.8 (0.78 – 4.27)	0.163
Lung cancer	9 (6.8)	1.0 (0.48 – 2.15)	0.965	0.9 (0.43 – 1.92)	0.808
Depression	10 (7.6)	0.9 (0.43 – 2.06)	0.880	0.8 (0.39 – 1.81)	0.654
Medications					
Statins	56 (42.4)	1.3 (0.85 – 2.06)	0.215	1.4 (0.91 – 2.25)	0.121
Beta-blockers	57 (43.2)	0.9 (0.58 – 1.35)	0.566	0.9 (0.59 – 1.39)	0.643
GER medication	13 (9.8)	0.9 (0.48 – 1.71)	0.752	1.2 (0.64 – 2.28)	0.556
ACE inhibitors or angiotensin 1 antagonists	48 (36.4)	1.4 (0.94 – 2.04)	0.103	1.1 (0.70 – 1.66)	0.737
Anticoagulants	15 (11.4)	1.1 (0.59 – 1.89)	0.849	0.7 (0.39 – 1.37)	0.332
Platelet function drugs ^d^	58 (43.9)	1.2 (0.77 – 1.88)	0.420	1.3 (0.82 – 2.04)	0.277
Inhaled corticosteroids	12 (9.1)	1.4 (0.71 – 2.83)	0.327	1.0 (1.03 – 1.07)	0.290
Antidiabetic drugs ^e^	22 (16.7)	1.6 (0.88 – 2.84)	0.123	1.7 (0.94 – 3.03)	0.082
Insulin	9 (6.8)	2.4 (0.95 – 6.24)	0.063	2.8 (1.07 – 7.09)	0.036
Thyroid medication	16 (12.1)	0.9 (0.49 – 1.81)	0.858	1.0 (0.50 – 1.90)	0.946
Allopurinol	4 (3.0)	0.8 (0.18 – 3.21)	0.717	0.6 (0.14 – 2.37 )	0.439

*N* number, *SD* standard deviation, *95 % C I* 95 percent confidence interval, *HRCT* high resolution computed tomography, *UIP* usual interstitial pneumonia, *TIA* transient ischemic attack, *OSA* obstructive sleep apnea, *GER* gastro-esophageal reflux, *COPD* chronic obstructive pulmonary disease

^a^Adjusted for age, gender and smoking history and DLco% or CPI

^b^Coronary artery disease, hypertension and cerebral infarction

^c^Heart failure for any reason

^d^Acetylsalicylic acid, dipyridamole and clopidogrel

^e^Metformin, glimepiride and sitagliptin

Comorbidities had been most often diagnosed before IPF (Fig. 2). CVDs and CAD were more often diagnosed before IPF in ex-smokers compared to current smokers (p <0.001, for both) and non-smokers (p=0.011 and p=0.016, respectively). CVDs were also more likely to be diagnosed before IPF in non-smokers than in current smokers (p=0.044) and in non-smoking males in comparison to male ex-smokers (p=0.002). Non-smokers (p=0.046) and ex-smokers (p=0.001) had received a GER diagnosis more often before the diagnosis of IPF compared to current smokers (p=0.046 and 0.001, respectively). After adjustment for age, smoking status, gender and separately both DLco % and CPI, it was found that the time of the comorbidity diagnosis in relation to the IPF diagnosis did not have any effect on survival.

Pharmacological treatments for IPF are presented in Table 6, symptomatic medical treatment in Table 7 and medications for comorbidities in Table 8.

**Table 6:** Pharmacological treatment of IPF in the years 2002 – 2016^a^

Medication	N / %
No medication	42 / 31.8
Total	
Corticosteroids	75 / 56.8
Azathioprine	28 / 37.2
N-acetylcysteine	30 / 40.0
Cyclophosphamide	16 / 12.1
Pirfenidone	6 / 4.5
Nintedanib	3 / 2.3
Mycophenolate mofetil	1 / 0.8
Single medication	
Corticosteroids	24 / 18.2
N-acetylcysteine	11 / 8.3
Pirfenidone	5 /3.8
Nintedanib	1 /0.8
Triple therapy ^b^	7 /5.3
Combined with prednisone as only medication	
Azathioprine	14 / 10.6
N-acetylcysteine	13 / 9.8
Cyclophosphamide	8 / 6.1
After prednisone therapy	
Nintedanib	2 /1.5
Before triple therapy	
Cyclophosphamide + Corticosteroids	2 / 1.5
After triple therapy	
Pirfenidone	1 / 0.8
Cyclophosphamide	3 / 2.3
Mycophenolate	1 / 0.8

^a^Diagnosis of IPF between years 2002 – 2012, while the follow-up information of medication was gathered until 16.10.2016. Reimbursement (by KELA) in Finland for pirfenidone in 1^st^ June 2013 and for nintedanib in 1^st^ December 2015.

^b^Triple therapy = azathioprine, N-acetylcysteine and prednisone

**Table 7:** Symptomatic medical treatment for IPF

Medication	N / %
Opioids ^a^	27 / 20.5
Oxygen therapy	45 /34.1
Ambulatory	17 / 37.7
Long term	28 / 21.1
Inhaled corticosteroids	9 / 6.8
Short-acting beta-agonists	7 / 5.3
Anticholinergic drugs	2 / 1.5
Theophylline	1 / 0.8
Montelukast	1 / 0.8
Mycolytics	3 / 2.3

^a^ Oxycodone, Fentanyl

**Table 8:** Medications for comorbidities

Medication	N (%)
Statins	56 (42.4)
Beta-blockers	57 (43.2)
ACE inhibitors or angiotensin 1 antagonist	48 (36.4)
GER medication	13 (9.8)
Allopurinol	4 (3.0)
Anticoagulants	15 (11.4)
Antiplatelet therapy ^a^	58 (43.9)
Inhaled corticosteroids	12 (9.1)
Antidiabetic drugs ^b^	22 (16.7)
Insulin	9 (6.8)
Thyroid medication	16 (12.1)

*N* number of patients with the medication

^a^Acetylsalicylic acid, dipyridamole and clopidogrel

^b^Metformin, glimepiride and sitagliptin

## Discussion

The smoking habits in conjunction with gender, clinical characteristics and comorbidities were studied in this retrospective real-life IPF population revealing that ex-smokers had a shorter survival time compared to non-smokers and current smokers. Furthermore, current smokers were significantly younger at diagnosis and at death than non-smokers and ex-smokers, but no differences in the major comorbidities i.e. CVD, CAD, hypertension, diabetes were found in relation to smoking history. Current smokers, who had smoked for more years and had more pack-years than ex-smokers, had more smoking-associated comorbidities such as COPD and lung cancer.

The results of our study confirm the findings of the previous studies in which unadjusted survival time was longer in current smokers and in non-smokers than in ex-smokers and similarly severity adjustment eliminated the survival advantage [2, 3]. We also observed that at the time of IPF-diagnosis, current smokers were significantly younger than either non-smokers or ex-smokers, a finding in agreement with several published studies [2, 3]. When compared to previous studies, however, ex-smokers and non-smokers were remarkably older but had better preserved lung function [1–3]. In our study however, despite the younger age of current smokers, the PFT and CPI did not differ between the groups of different smoking histories, although current smokers had smoked significantly more years and pack-years compared to ex-smokers. When comparing the severity adjusted survival of current smokers and ex-smokers, there was still a marginal non-significant survival advantage for current smokers but this became diminished when age was added into the model. This may be due to under-powering i.e. the low number of patients, but it is however noteworthy that the proportion of current smokers was higher (13%) in our study than in the publication of Antoniou et al. (8%) in which a survival difference was abolished when CPI was incorporated into the severity adjustment [2].

Differences in the proportions of current smokers, non-smokers and ex-smokers can be seen in published studies from different countries. In our study, 35.2% of the patients with IPF were non-smokers. In the Finnish IPF register, in which the patients are collected prospectively including patients from the year 2012 onwards, as many as 44% of the patients were non-smokers [23]. However, the registry includes only patients who have provided consent to participate, meaning that the IPF patients with rapidly progressing disease or older age and with cognitive impairment have not been taken into account. The percentage of non-smokers is surprisingly different in these Finnish studies compared to the results of one other Nordic country, i.e. Denmark, in which only 19% of the patients were non-smokers in a similar size of cohort (n=121) as ours [10]. This may be due to cultural differences and also the reason why the “healthy smoker effect” was not displayed in our study. The “healthy smoker effect” is a term originating from COPD studies; it has been used to explain why current smokers exhibit milder disease than non-smokers and ex-smokers in terms of PFT, DLco and CPI [2, 24]. This effect was not confirmed in our IPF population.

King et al. claimed that current smokers had a longer survival than ex-smokers [3]. Current smokers were younger compared to those with other smoking histories which meant that age was found to be a significant predictor of survival [3]. This study disagrees with the study of King et al. since ex-smokers and non-smokers were remarkably older at the time of diagnosis and furthermore, no significant differences in the PFT and DLco results were observed. The reason why non-smokers and ex-smokers were about 10 years older at the time of diagnosis in this study compared to previous studies is uncertain but may partly explain the reason why current smokers exhibited a longer survival [2, 3].

A more recent study comparing non-smoking IPF patients to a combined group of ex-smokers and current smokers confirmed our results of non-smokers being mostly female, but found no difference in age between the groups [4]. In that particular study, non-smokers had significantly worse survival, lower DLco and higher CPI and even adjusted for CPI, the poorer prognosis of non-smokers was retained and baseline CPI, DLco% and FVC % were not statistically significantly predictive for survival, a finding which is different from that of Antoniou’s et al. [2, 4].

In our study, the HRCT classification was performed according to the present guidelines [22]. We found that the most common pattern in HRCT in non-smokers was definite UIP in agreement with previous reports [4, 22]. Our results are also in line with the previous publications showing that non-smokers accounted for 35.4 % of the definite UIP patterns in HRCT. Furthermore, the gender distribution between the different radiological categories was similar as in other reports [25, 26].

The prevalences of GER, lung cancer, COPD, hypertension, CAD and diabetes were in line with most previous publications, although OSA was less frequent (4.5 %) in our cohort [6, 7, 27]. Our results support the earlier findings that CVDs are the most common comorbidities experienced by IPF patients [7, 28]. It has been reported that the overall number of comorbidities is significantly associated with survival in IPF, but this was not the case in our patients [6]. IPF patients with CAD have been reported to have worse outcomes [12]. If CVDs were diagnosed after the diagnosis of IPF, this was associated with increased mortality in IPF as was also the presence of either diabetes or thyroid disease [11]. In contrast, in our study, diabetes, thyroid medication and CAD alone did not influence survival, and furthermore, the CVDs and CAD had been most often diagnosed before the diagnosis of IPF. This may be caused by the high prevalence of CVD and CAD in eastern Finland [29]. However, the diagnosis of any CVD and COPD at any time and the use of insulin medication at the time of diagnosis were related to poorer survival in the severity adjusted analyses. Our results revealed no effect of statin and anticoagulant medication on survival in IPF unlike previously published investigations [6, 16–19]. The use of GER medication and a GER diagnosis have been associated with longer survival times in some previous investigations, but here, they exerted no effect on survival [6, 15].

In this study cohort, female IPF patients had more asthma and diabetes than their male counterparts. Current smokers had more COPD and lung cancer compared to ex-smokers probably due to the fact that current smokers had smoked more than ex-smokers in terms of both years and in pack-years. When comparing the subgroups with different smoking histories, surprisingly, no statistically significant differences were detected in the proportion of patients with CVD, CAD or GER as comorbidity. In this study, we could not prove that smoking history had any significant effect on major comorbidities or their prevalence, and even although older age seemed to add to the number of comorbidities, the quality and severity of the comorbidity were probably more important than the actual number of comorbidities.

## Conclusions

It can be speculated that smoking may, at least partly, influence the onset of IPF since current smokers developed the disease earlier and died at a younger age than either non-smokers or ex-smokers. No significant differences in the major comorbidities were found between IPF patients with different smoking histories.

## Abbreviations

ATS: American Thoracic Society
CAD: coronary artery disease
COPD: chronic obstructive pulmonary disease
CPI: composite physiologic index
CVD: cardiovascular disease
DLco: diffusion capacity of carbon monoxide
DLco/VA: potential value of diffusion capacity per liter of lung volume
ERS: European Respiratory Society
FEV1: forced expiratory volume in one second
FEV1/FVC: forced expiratory volume in one second and forced vital capacity ratio
FVC: forced vital capacity
GER: gastro-esophageal reflux
HRCT: high resolution computed tomography
ICD-10: International Classification of Diseases version 10
IPF: Idiopathic pulmonary fibrosis
PFT: pulmonary function tests
UIP: usual interstitial pneumonia
